# Mini-Implant Insertion Using a Guide Manufactured with Computer-Aided Design and Computer-Aided Manufacturing in an Adolescent Patient Suffering from Tooth Eruption Disturbance

**DOI:** 10.3390/bioengineering11010091

**Published:** 2024-01-18

**Authors:** Christina Weismann, Kathrin Heise, Maite Aretxabaleta, Marcel Cetindis, Bernd Koos, Matthias C. Schulz

**Affiliations:** 1 Department of Orthodontics, University Hospital Tuebingen, Osianderstr. 2-8, 72076 Tuebingen, Germanymaite.aretxabaleta-santos@med.uni-tuebingen.de (M.A.);; 2 Department of Oral and Maxillofacial Surgery, University Hospital Tuebingen, Osianderstr. 2-8, 72076 Tuebingen, Germanymatthias.schulz@med.uni-tuebingen.de (M.C.S.)

**Keywords:** impaction, tooth germ displacement, orthodontic, skeletal anchorage, transversal maxillary expansion, cone beam computed tomography, surgical guides, orthodontic implants, temporary anchoring device

## Abstract

Due to dental diseases, anatomical restrictions, and mixed dentition, the reduction in the number of teeth and the displacement of tooth germs pose challenges in orthodontic treatment, limiting anchorage options. The presented case demonstrates an advanced treatment solution using digital CAD/CAM-technologies and medical imaging for the creation of a mini-implant template. A 12-year-old male patient experiencing delayed tooth eruption, multiple impacted germs, and maxillary constriction underwent intraoral scanning and CBCT. Utilizing coDiagnostiX^TM^ Version 10.2 software, the acquired data were merged to determine the mini-implant placement and to design the template. The template was then manufactured through stereolithography using surgical-guide material. Mini-implants were inserted using the produced appliance, enabling safe insertion by avoiding vital structures. Surgically exposed displaced teeth were aligned using a Hyrax screw appliance anchored on the mini-implants for rapid palatal expansion (RPE) and subsequently used as fixed orthodontics to align impacted teeth. The screw was activated daily for 10 weeks, resulting in a 7 mm posterior and 5 mm anterior maxillary transversal increase. Skeletal anchorage facilitated simultaneous RPE and tooth alignment, ensuring accuracy, patient safety, and appliance stability. The presented case shows a scenario in which computer-aided navigation for mini-implant positioning can enhance precision and versatility in challenging anatomical cases.

## 1. Introduction

Tooth eruption is an important factor in maxillofacial development during adolescence. The occurrence of ectopic tooth eruption and the subsequent changes in its eruption path might lead to malocclusion or dental anomalies [1,2]. This results in a positional change between the maxilla and mandibula. Additionally, an adaption of the stomatognathic system can occur owing to the structural changes induced during the growth process. This might lead to skeletal malformation [3]. The eruption disturbance can arise from systemic factors, e.g., sex, age, ethnicity, genes, or a primary failure of eruption syndrome (PFE) [4]. The situation can be further aggravated by the presence of other physical obstruction factors like cysts [5], supernumerary teeth, ectopic eruptions, ankylosis [6], traumas/surgeries [7], or mucosal barriers [8]. Many different terms are currently used in the literature to describe disturbances and disorders in tooth eruption, which are mostly referred to as “impaction” and “retention”. Impaction is defined as “the cessation of eruption caused by a radiographically or clinically detectable physical barrier in the eruption pathway such as supernumerary tooth buds” [9]. Among other possible reasons, a lack of space from crowding, premature loss of deciduous teeth, or abnormal tooth germ positions can contribute to impaction [10]. Meanwhile, retention can be further categorized as being primary or secondary. Primary retention is defined as the absence of a physically identifiable barrier as an explanation for the cessation of a normally developed and placed tooth germ before its emergence [6,11]. Meanwhile, secondary retention is understood as the cessation of eruption after emergence without any physical barrier and as a result of an abnormal position [11,12]. Although often used synonymously, the etiologies of impaction and retention differ and, thus, require different treatments. Considering that tooth eruption is a multifactorial process, a deficiency in one factor might be compensated for by another [13]. However, if compensation fails, the process might be disturbed, potentially leading to a delayed tooth eruption (DTE), significantly deviating from the norm [14].

An early diagnosis of tooth eruption disturbances is crucial for initiating orthodontic treatment promptly, preventing delays and potentially simplifying therapy options. In cases of DTE with malpositioned germs, the reduced availability of teeth for dentition-based anchorage complicates the orthodontic treatment planning. As deciduous teeth become mobile and permanent teeth have difficulties in erupting due to space constraints [15], the use of a dentoalveolar anchorage with removable or fixed appliances is challenging. A skeletal anchorage, specifically using mini-implants placed intraorally with minimal surgical invasiveness, offers a feasible alternative in such cases [16]. This anchorage type can be combined with rapid maxillary expansion (RPE) techniques like the hybrid Hyrax, forming micro-implant-assisted RPE (MARPE) [17,18,19]. MARPE can ensure a correct skeletal expansion and a lateral translation of the underlying maxillary bone through the application of expansion forces closer to the maxilla’s center of resistance [20]. Furthermore, it minimizes the dentoalveolar effects, avoiding a buccal tipping of the posterior teeth [21], and eliminates the need for an attachment to the first molars.

However, challenges arise in determining the optimal position for maxillary mini-implants in patients with multiple ectopic and malpositioned germs in order to avoid injury of the impacted germs. In such cases, three-dimensional medical imaging technologies, e.g., cone beam computed tomography (CBCT) [22], in combination with computer-aided design and computer-aided manufacturing (CAD/CAM) technologies, present a feasible solution for the digital planning of mini-implant insertion. This approach enables us to determine the optimal position, angulation, and lengths of mini-implants pre-surgically, thus enabling a risk-free and individually skeletal anchorage [23]. This planning can be transferred into the clinical situation using a completely CAD/CAM designed and CAD/CAM manufactured drilling template [24], aiming to decrease the risks of potential complications during surgery while increasing the stability and efficiency of the mini-implants.

The presented case describes an advanced therapy approach of applying a mini-implant drilling template in an adolescent patient with multiple ectopic tooth germs, DTE, and a reduced dentition. This innovative method uses three-dimensional planning and CAD/CAM technologies, representing a significant advantage for treating anatomically challenging situations. This case aims to demonstrate the application of an innovative treatment method under special clinical conditions. To the best of the authors’ knowledge, it is the first depiction of this treatment in a patient with multiple impacted tooth germs using a completely digitally planned and manufactured drilling template.

## 2. Clinical Example Presentation

### 2.1. Patient Case

**Figure 1:**
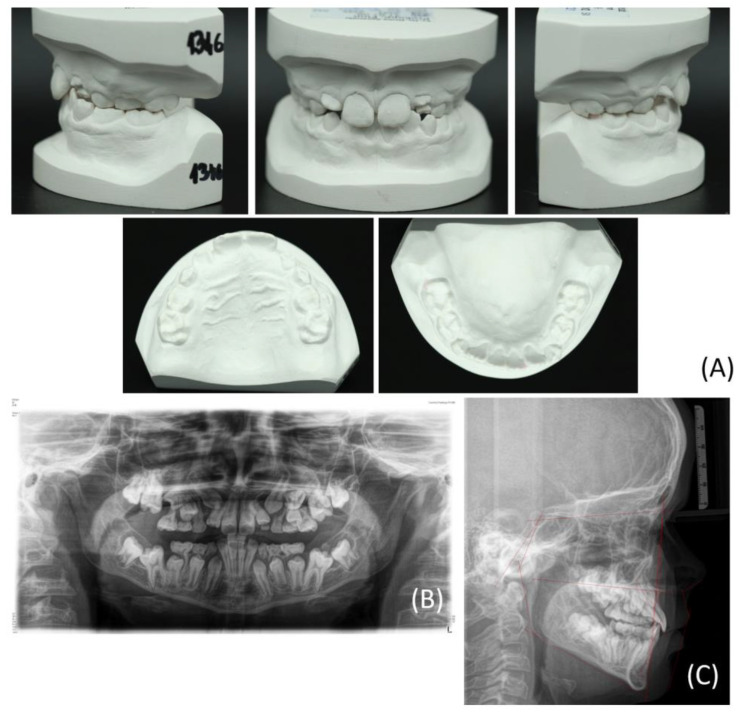
The images show a twelve-year-old patient showing a delayed tooth eruption of the permanent teeth, a disturbance in the eruption of the first molars, a displacement of multiple tooth germs, and a crowding, certainly of the canines and premolars in the maxilla. (**A**) Lateral, frontal, and top views of the maxillary and mandibular dental plaster casts of the patient at the time of initial presentation; (**B**) Panoramic radiograph revealing multiple dislocated tooth germs; (**C**) Lateral cephalogram depicting the skeletal growth pattern.

**Figure 2:**
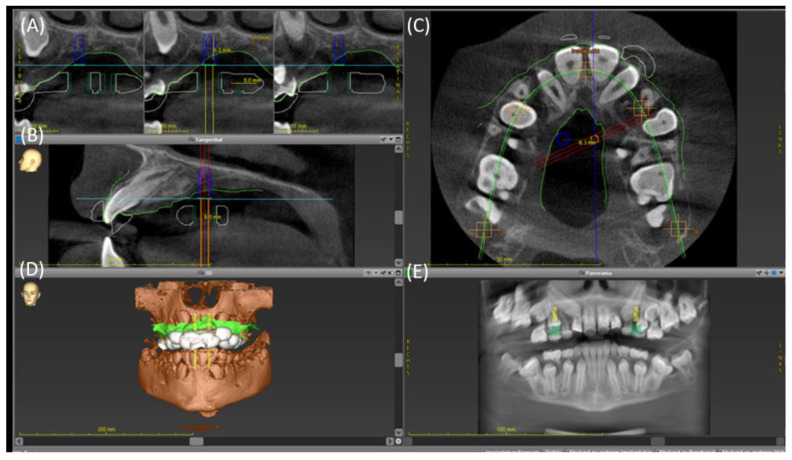
Screenshot of the planning of the two palatal mini-implants using the coDiagnostiX^TM^ (Dental Wings GmbH, Chemnitz, Germany). (**A**) The planned implant position in the coronal plane showing the vertical bone dimension of the hard palate. (**B**) Sagittal plane of the right implant ensuring a sufficient distance to the root of the inclined lateral incisor. (**C**) Transversal plane of the maxilla displaying the multiple retained tooth germs restricting the region for safe mini-implant insertion. (**D**) Three-dimensional depiction of the bone (brown), the intraoral maxillary soft tissue scan (green), and the final design of the drilling template (white). (**E**) Panoramic radiograph-like view of the case displaying the planned mini-implant positions.

A 12-year-old male patient presented in the Department of Orthodontics at University Hospital Tübingen. He was referred by a registered orthodontic colleague. The medical history revealed a hearing impairment with incorporated hearing devices. Furthermore, no regular medication or allergies were declared. The clinical picture revealed a skeletal class III configuration, an Angle class III malformation with a crossbite on the left side, and a maxillary constriction of 4 mm in the posterior region. The mandible had a deviation of 1.5 mm to the left side. The patient showed a DTE of the succedaneous teeth by having a dental age of 7 years. Initial findings revealed that the erupted teeth were 11, 21, and 32–42, with 12 and 22 erupting in an inclined malposition. The deciduous canines and molars were still in situ. The 6-year molars were not erupted, showing a displacement tendency, certainly in the mandible. A medial diastema of 1 mm was visible (Figure 1; Table 1). The screening of the temporomandibular joint was without any pathological findings. The mouth opening distance measured between the front teeth was 40 mm. The initial panoramic radiograph revealed displaced multiple tooth germs and a crowding of the canines and premolars in the maxilla (Figure 1B). The tooth germ crown 13 was located close to the apex of tooth 12 (Figure 2B). Furthermore, the erupted permanent front teeth were improperly inclined and angulated. No mechanical interference was evident, particularly in the posterior region. Additionally, a PFE was genetically disclosed before the beginning of the orthodontic treatment.

### 2.2. Treatment Methods and Objectives

Based on the clinical and radiographic findings, the following treatment plan was defined:

1. Surgical exposure of the displaced and impacted teeth.
2. RPE using a skeletal-anchorage Hyrax appliance.
3. Resolution of the crossbite through maxillary transversal expansion.
4. Alignment of the surgically exposed teeth by using skeletal anchorage, compensating for insufficient dentoalveolar support due to the reduced dentition.
5. Eruption control of the displaced permanent teeth after dental arch expansion.
6. Correction of the angulation, inclination, and rotation of the malpositioned teeth using a fixed orthodontic appliance.

### 2.3. Design and Manufacturing of the CAD/CAM Drilling Template

#### 2.3.1. Digital Planning and Template Design

At the first appointment, an intraoral scan (IOS) (Trios 3^®^ scanner, 3Shape A/S, Copenhagen, Denmark) of the maxilla was performed. Additionally, a CBCT scan was carried out in order to assess the three-dimensional location of the tooth germs. The data of the maxillary IOS data were saved in standard tessellation language (STL) format, and the CBCT images were saved as Digital Imaging and Communication in Medicine (DICOM) format. Both data sets were imported into the coDiagnostiX^TM^ software Version 10.2 (Dental Wings GmbH, Chemnitz, Germany), a software commonly used for planning dental implants. The data were merged using a semi-automatic superimposition method. Subsequently, the optimal position of the mini-implants in the palatal area was determined by an experienced orthodontist and oral surgeon. Therefore, the challenging anatomical conditions, e.g., the displaced tooth germs and the thickness of the palatal bone had to be considered. For the anchorage of the mini-implants, a region with a sufficient bone volume and an adequate distance to the tooth buds had to be located. Thus, the region of interest was analyzed in the coronal, sagittal, and transversal planes (Figure 2A–C). After determining the optimal position for the two palatal mini-implants, a tooth-borne template was virtually designed (Figure 2). Subsequently, the designed drilling template was exported as an STL file.

#### 2.3.2. Manufacturing of the Drilling Template

**Figure 3:**
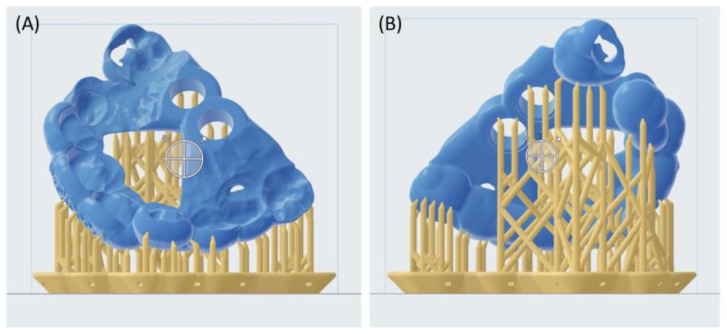
Placement of the customized drilling template on Preform software (Formlabs), considering the frontal (**A**) and posterior (**B**) views. The support structures were not placed in areas of higher-accuracy interest, such as the negative of the maxillary scan and holes of the implants.

The template was manufactured by using CAM technology, specifically using additive manufacturing with a stereolithography (SLA) vat polymerization machine (Form3B, Formlabs, Sommerville, MA, USA). The template was imported into Preform V3.18 (Formlabs, Sommerville, MA, USA), where it was positioned on the printing platform as shown in Figure 3. The support structures were automatically calculated using the default set-up (full raft, thickness 1 mm, touchpoint size 0.6 mm). The inclination of the template in the plotter and the positioning of the support structures were then manually adjusted to preserve accuracy in critical areas, such as holes corresponding to teeth and implants. A European Medical Device Regulation (EMDR) class I, biocompatible, and autoclavable material specifically for application in surgical guides (Surgical guide material, Formlabs) was used with a layer height of 100 µm. The layer height was defined based on previous accuracy studies [26].

#### 2.3.3. Post-Processing of the Drilling Template

The post-processing followed the manufacturer’s instructions: washing for 20 min with 98% IPA in Formwash (Formlabs), drying with compressed air, and post-curing for 30 min at 70 °C in Formcure (λ = 405 nm, Formlabs). After complete polymerization, the support structures were removed, and the surface marks were erased through manual burnishing and polishing.

#### 2.3.4. Supplementary CAM of a Model Cast

**Figure 4:**
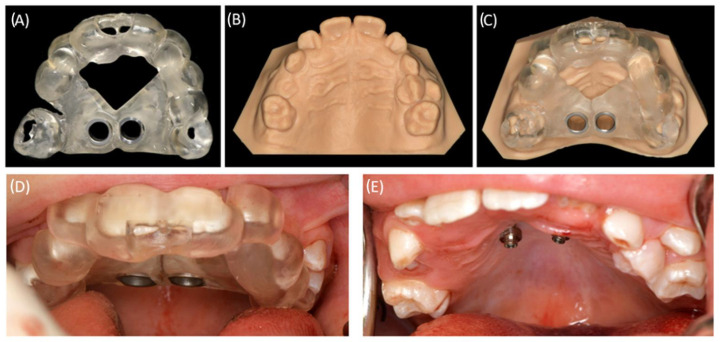
CAD/CAM-based implant drilling template procedure. (**A**) CAD/CAM manufactured tooth-borne template. (**B**) Three-dimensional-printed maxillary model. (**C**) In vitro check of the fit of the custom-made template on maxillary model. (**D**) Intra-operative placement of the template in the maxilla. The incisal fenestration on teeth 11 and 21 enables an intra-operative fit check of the template. (**E**) Intra-oral view following successful guided placement of the mini-implants.

In order to ensure the fitting of the manufactured appliance in vitro (Figure 4), a model of the maxilla was designed using an appliance designer software (3Shape) and produced using the Form3B SLA machine (100 µm layer height, Model V2.0. material, Formlabs). The model was positioned in Preform (Formlabs) at an inclination, opposite the occlusal plane to the printing platform side. The support structures were automatically generated. Again, the post-processing followed the manufacturer’s instructions (Formwash: 10 min IPA 98%, Formcure: 60 min 60 °C). Finally, the support structures were removed after complete polymerization.

### 2.4. Treatment of the Clinical Example

#### 2.4.1. First Surgical Exposure of the Impacted Teeth and Insertion of the Mini-Implants

**Figure 5:**
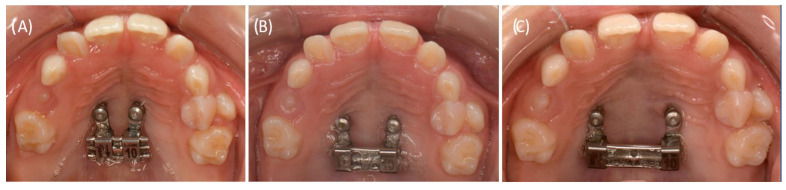
Intraoral pictures depicting the increase in the transversal maxillary dimension by the combination of the mini-implants and the Hyrax screw at different time points: (**A**) before activation, (**B**) after four weeks of the activation period, and (**C**) after ten weeks of activation.

Teeth 37, 36, 46, and 47 were surgically exposed in general anesthesia. Additionally, the two palatal mini-implants (2 × 9 mm, Benefit System; PSM North America, Indio, CA, USA) were inserted without predrilling using the previously manufactured template (Figure 4). A contra-angle screwdriver was applied for the mini-implant insertion. After surgery, the bmx DIRECT Hyrax screw (10 mm, BENEfit^®^-System, Dentalline, Birkenfeld, Germany) was fixed to the mini-implants to achieve a MARPE (Figure 5).

#### 2.4.2. RPE Using the Skeletal-Anchorage Hyrax Appliance

The total activation time of the Hyrax screw was approximately ten weeks in which the parents were instructed to perform the daily activation. During this period, an increase of seven millimeters in the posterior region (deciduous molars) and five millimeters in the anterior region (deciduous canines) could be achieved for the maxillary transversal expansion. The increase in the medial diastema during the activation period indicated the skeletal transversal expansion of the maxilla (Figure 5). After the activation phase, the crossbite was resolved as a result of the transversal expansion.

#### 2.4.3. Alignment of the Surgically Exposed Teeth by Using Skeletal Anchorage

The Hyrax appliance served as a retention tool for the transversal maxillary expansion and as a skeletal anchored appliance for aligning the impacted teeth by using a cantilever. For the latter, the Hyrax appliance was modified in the dental laboratory to provide fixed attachments for the cantilevers’ wires.

**Figure 6:**
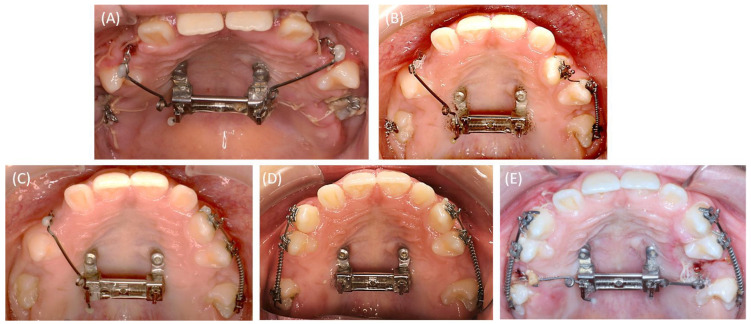
Intraoral images depicting the modification of the Hyrax appliance for skeletal anchorage to align the impacted teeth: (**A**) immediately after the surgical exposure of the teeth (16, 13, 23, 26) with the Hyrax modification and sutures still in situ; (**B**) alignment of tooth 23 into the dental arch using a fixed orthodontic segment appliance; (**C**) continued alignment of the dental arch; (**D**) alignment of tooth 13 into the dental arch and the use of open coils in regions 15 and 25 attached to the fixed orthodontic appliance; (**E**) after the third surgical exposure and alignment into the dental arch using cantilevers of the teeth 15 and 25 with the Hyrax device as skeletal anchorage.

**Figure 7:**
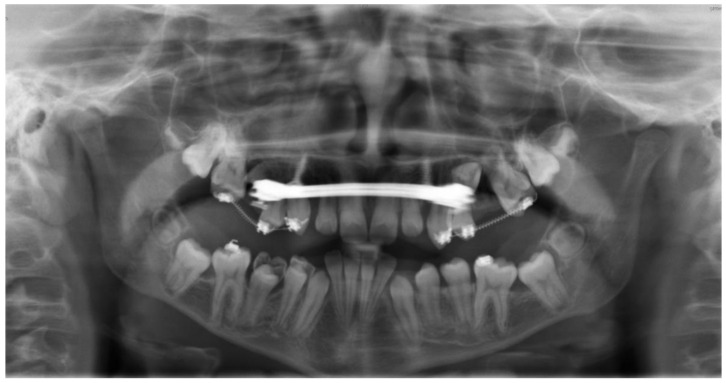
The follow-up panoramic radiograph at the age of 13 years reveals the displaced germs of teeth 15 and 25, partially superimposed by the cantilever structure in the palatal area. Both mini-implants show no sign of peri-implant osteolysis.

In order to align teeth 13, 16, 23, and 26, a secondary surgical exposure was performed. Two cantilevers (0.017 × 0.025-inch TMA wire, Dentaurum, Ispringen, Germany) were used for the orthodontic alignment of the permanent maxillary canines. Following the surgical exposure, a fixed orthodontic appliance segment was placed for the alignment of the dental arch (Figure 6B, second quadrant). After a six-month treatment period, the surgically exposed teeth were properly aligned (Figure 6). Additionally, two open coils in the region of second premolars 15 and 25 were employed to expand the space for alignment. After the expansion of the dental arch, teeth 15 and 25 were exposed in a third surgical intervention. Both premolars were aligned into the dental arch using two cantilevers (0.017 × 0.025-inch TMA wire) (Figure 6E and Figure 7). The final result is depicted in the follow-up panoramic radiograph in Figure 7, where both mini-implants exhibit no sign of peri-implant osteolysis. Throughout the 22-month treatment period, no unwanted side effects were observed by the treating orthodontist or reported by the patient.

## 3. Discussion

The current clinical example shows a possibility for applying a customized and CAD/CAM manufactured template for the insertion of mini-implants in a patient suffering from multiple displaced ectopic tooth germs and DTE. The authors are unaware of similar clinical cases in the current literature. For this particular example, the challenge centered around the necessity for maxillary expansion, the surgical exposure of multiple teeth, and the subsequent integration of orthodontic alignment treatment. A complicating factor was the reduced number of teeth in the oral cavity available for a tooth-borne orthodontic appliance. Consequently, a non-dentoalveolar and maximally anchored appliance was required to orthopedically expand the maxilla and to ensure sufficient stability for the alignment of the ectopic teeth.

The presence of multiple malpositioned tooth germs in the maxilla posed a challenge in identifying a secure and low-risk anatomical site for the insertion of mini-implants [24,27]. The accurate placement is crucial to ensure a prolonged stability of the Hyrax screw appliance during the orthodontic treatment. Thus, the risk of potential complications, e.g., anchorage loss, instability of implants, mini-implant fatigue fractures, inflammation of adjacent tissue or appliance failure [28,29], can be reduced. Given the potential risk of injuring erupting teeth or unerupted germs, the alveolar process was not considered as suitable for mini-implant insertion. Furthermore, the literature reports a higher rate of implant loss and failure in this region [30]. Alternatively, the retromolar region would be a possible option. However, this region was subsequently ruled out due to inadequate preconditions, e.g., thick, soft tissue and insufficient bone quality [31]. Consequently, the anterior palatal region was considered as the most suitable area for implant insertion in this case [32], despite the heightened risk of tooth germ injury, certainly for the displaced maxillary canines. The present clinical example showed favorable hard and soft tissue conditions, with adequate vertical bone dimension and bicortical laminae, enabling a sufficient stability of the mini-implants [25,33,34,35]. No crucial anatomical structures in this area could be detected, and the soft-tissue conditions of the hard palate offer favorable peri-implant conditions, ensuring an easy accessibility for oral hygiene and device activation [36]. Furthermore, the palatal area is not in the aesthetic zone [37] and is accessible for minimal-invasive anchorage approaches, particularly when a dentoalveolar anchorage would be insufficient. Additionally, the region enables chairside procedures for fixing the appliance on the mini-implants without requiring additional local anesthesia [38]. These conditions enable a wide range of non-invasive anchorage strategies for the necessary tooth movements. Furthermore, the literature frequently highlights the advantages of a skeletal fixed appliance compared to a removable one [39,40,41,42].

In the radiographic diagnostic work-up, a CBCT is recommended as the golden standard for patients suffering from multiple ectopic tooth germs and DTE. The three-dimensional radiograph might provide comprehensive information considering the localization of tooth germs, potential supernumerary teeth, or severe root resorptions [43]. This precision is critical to establish an accurate diagnosis and is the base for a sound treatment planning and, thus, a successful therapy. In the presented case, a CBCT was used for the combined orthodontic and surgical treatment planning, justifying its heightened radiation exposure compared to a two-dimensional panoramic radiograph in challenging patient cases.

The computer-aided static navigation technique of using CBCT not only facilitates the pre-surgical interdisciplinary discussion on the orthodontic treatment plan but also enhances the accuracy in the planning of mini-implant insertion [44,45,46]. In the presented example, the knowledge of the accurate positions of the impacted tooth germs using CBCT imaging allowed for the determination of the position and insertion depth of the mini-implant. Furthermore, guided implant insertion improved the control over mini-implant angulation and parallelism. Thus, the stability of the maxillary skeletal expansion appliance through a bicortical anchorage and the optimal orientation might be increased [47,48]. Therefore, a CBCT-based treatment planning can be considered superior to panoramic radiography or lateral cephalography when mini-implant placement is required.

The described clinical example depicts a challenging scenario characterized by a skeletal class III configuration and crowding of tooth germs in the maxilla. Consequently, there was the necessity for maxillary expansion to enlarge the dental arch and enable an intrinsic tooth eruption. The defined treatment plan comprised not only the orthodontic intervention but also the requirement for the surgical exposure of the ectopic teeth to enable orthodontic tooth movement. However, using conventional orthodontic appliances was not possible due to the reduced dentition. The increasing popularity of orthodontic mini-implants, reducing the need for dentoalveolar anchorage, has led to the development of various MARPE techniques customized according to individual treatment needs [49]. In this clinical example, the use of a Hyrax appliance proved to be adequate. Firstly, it facilitated the maxillary skeletal expansion without requiring additional dentoalveolar anchorage such as at the first molars as in a hybrid Hyrax. This versatility makes the appliance suitable for adolescent and adult patients with a periodontally compromised dentition. Additionally, potentially related complications and unwanted dental side effects are not likely [20,50]. Furthermore, the proposed approach was used for the simultaneous retention and adjustment of the displaced teeth by working as a skeletal anchored appliance. The Hyrax device was placed immediately after mini-implant insertion in a single appointment when the compliance of the patient was given. Moreover, the high primary stability allowed for the loading of the mini-implants with the Hyrax appliance without the need for achieving osseointegration, enabling an immediate treatment to begin [34].

The clinical example presented demonstrates the feasibility and advantages of the intra-operative use of a drilling template for the mini-implant insertion. This approach simplifies the surgery, particularly in cases with limited space and a restricted field of view, allowing for more time-efficient procedures and reducing the length of general anesthesia. Furthermore, the use of drilling templates might support the education of inexperienced clinicians [49].

A complete digital workflow, using IOS and CBCT data for computer aided design and manufacturing of a patient-specific medical appliance seem to offer advantages such as feasibility, efficiency, and ease of implementation in daily clinical practice in comparison to conventional approaches. Despite the potential barriers to digital technology adoption, including initial costs and the associated learning curve [51], the benefits counterbalance it by providing a decreased human error and an increased patient safety [52,53].

A complete digital workflow is based on the use of medical imaging data for the design and manufacturing using CAD/CAM technologies of a patient-specific medical appliance. In the presented example, IOS and CBCT data were used for designing a patient-personalized mini-implant drilling template which was subsequently manufactured through additive manufacturing. The use of a complete digital workflow can offer different advantages in comparison to conventional approaches in challenging patients. The workflow is feasible, efficient, and easy to implement into the daily clinical routine. Additionally, the implementation of digital technologies is known to decrease human errors and, thus, can increase the patient safety [52,53]. Despite these advantages, the burden of the inauguration of digital technologies, e.g., initial investment costs and learning curve, might hamper its adaptation in certain facilities [51].

## 4. Conclusions

The following conclusions can be drawn from this communication:

- Computer-aided static navigation techniques, applying IOS and CBCT, support a patient-specific, accurate, and safe planning for orthodontic mini-implant insertion.
- CAD/CAM technologies for the design and manufacturing of the insertion template have proven to be a feasible and efficient solution that can be easily implemented in the clinical routine, even in challenging patient cases.
- The presented protocol using CAD/CAM-based drilling templates might be supportive in the education of inexperienced clinicians.
- The skeletal-anchored Hyrax appliance provides an effective treatment for orthodontic therapy in challenging cases with a reduced dentition, allowing for both maxillary expansion and dental arch alignment in a single approach.
- The simultaneous insertion of mini-implants and the Hyrax appliance in a single intervention can optimize the treatment process.

In summary, the described clinical example illustrates that applying an established method, such as digitally planned, designed, and manufactured drilling templates for mini-implants, is not only feasible but also effective in anatomically challenging clinical situations.

## Figures and Tables

**Table 1:** Summary of the orthodontic and cephalometric analysis findings of the patient before initiating orthodontic treatment, according to the Hasund method [25].

Measurement	Value before Treatment	Measurement	Value before Treatment
SNA (82 ± 3°)	83.3°	Interincisal angle 1–1 (131 ± 6°)	131.6°
SNB (80 ± 3°)	81.3°	OK1-NA (22.0 ± 3°)	30.1°
AND (indiv.)	4.8°	UK1-NB (25.0 ± 3°)	16.3°
SN-Pg (82 ± 3°)	80.7°	OK1O-NA (4.0 ± 2 mm)	3.9 mm
NS-Ba (130 ± 6°)	127.6°	UK1O-NB (4.0 ± 2 mm)	2.9 mm
NL-NSL (8.5 ± 3°)	9.9°	Overjet	3 mm
ML-NSL (32.0 ± 6°)	33.4°	Overbite	3 mm
ML-NL (23.5 ± 3°)	23.5°	Mandibular deviation	1.5 mm right side
Wits appraisal (−0.3 ± 0.3 mm)	−1.0 mm		

## Data Availability

The data presented in this study are available on request from the corresponding author.
